# Supplementary material for: Optimization of Protocols for the Induction of Callus and Plant Regeneration in White Clover (Trifolium repens L.)
Source: Int J Mol Sci. 2023 Jul 9;24(14):11260. doi: 10.3390/ijms241411260 (PMC10378747; doi:10.3390/ijms241411260)
Supplement: Supplementary file 1 [file ijms-24-11260-s001.zip › ijms-2477968-supplementary.pdf]

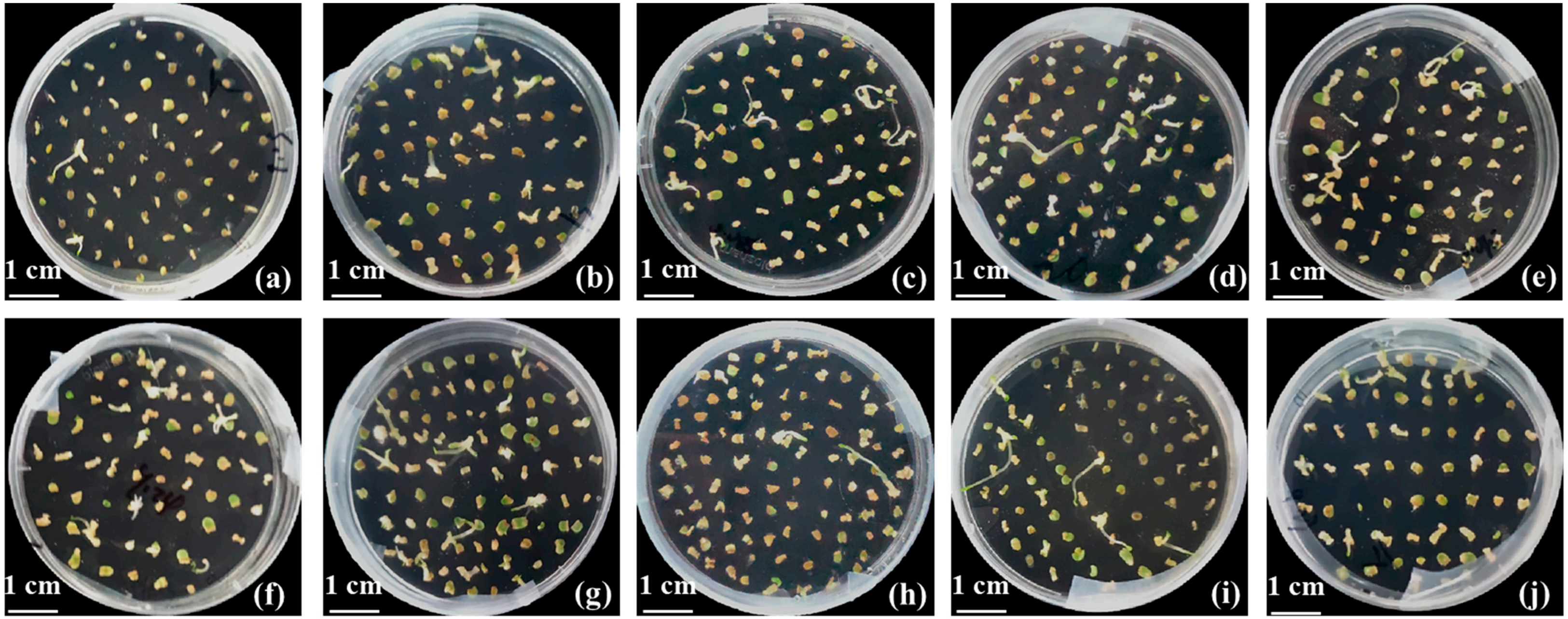

Supplement Figure S1. Calluses of different varieties of white clover at 20-days-old emerged from the cotyledon wounds. **(A)** Barbzan callus. **(B)** Koala callus. **(C)** HaHnony callus. **(D)** Ladino callus. **(E)** Sulky callus. **(F)** Haifa callus. **(G)** Miracle callus. **(H)** Pixie callus. **(I)** Zapican callus. **(J)** Mag callus.

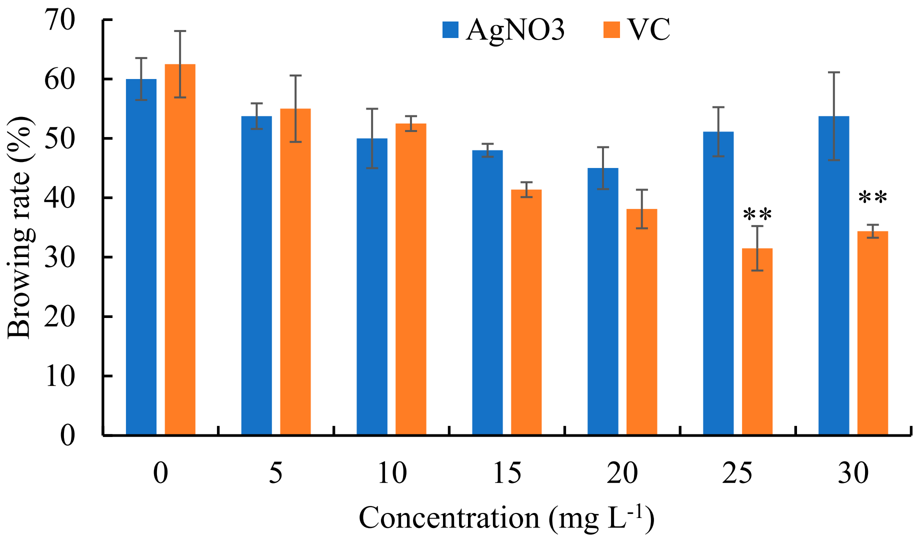

Supplement Figure S2. Comparison of $AgNO_3$ and VC for reducing browning rate at the same concentration gradient. "**" was significant at the 0.01 level.

Supplement Table S1. Names and origins of the tested white clover varieties**.**

Varieties	Amount	Seed source	Origin
Barbzan	1 kg	Evergeen International Co., Ltd.	Netherlands
Koala	1 kg	Evergeen International Co., Ltd.	Australia
HaHnony	1 kg	Evergeen International Co., Ltd.	America
Ladino	1 kg	Evergeen International Co., Ltd.	America
Sulky	1 kg	Evergeen International Co., Ltd.	Argentina
Haifa	1 kg	Beijing Hope Turf Techenogy Co., LTD	Australia
Miracle	1 kg	Evergeen International Co., Ltd.	America
Pixie	1 kg	Evergeen International Co., Ltd.	New Zealand
Zapican	1 kg	Beijing Hope Turf Techenogy Co., LTD	Argentina
MAG	1 kg	Beijing Hope Turf Techenogy Co., LTD	Argentina

Supplement Table S2. Transformation efficiency statistics using the leaf explant protocol.

**Method**	**Explants**	**Kan-resistant callus**	**Positive lines**	**Transformation Efficiency**
Article section 4.5	210	56	5(8.9%)	2.38%

Supplement Table S3. Culture medium composition for callus induction and differentiation

Chemica	CCM 1000ml	CDM1 1000ml	CDM2 1000ml
MS basal medium	4.74 g	4.74 g	4.74 g
Sucrose	3 g	3 g	3 g
Calcium gluconate	0.6 g	0.6 g	0.6 g
pH	5.8	5.8	5.8
Plant Aga	7 g	7 g	7 g
2,4-D stock solution (2 mg/ml)	1 ml	0	0
6-BA stock solution (0.5 mg/ml)	1 ml	2 ml	0
NAA stock solution (0.1 mg/ml)	0	-	1 ml

Supplement Table S4. Medium composition for white clover callus induction and proliferation

Chemica	CIM 1000ml	CM 1000ml	CCM-S 1000ml	CDM-S 1000ml	CRM-S 1000ml
MS basal medium	4.74 g	4.74 g	4.74 g	4.74 g	4.74 g
Sucrose	3 g	3 g	3 g	3 g	3 g
Calcium gluconate	0.6 g	0.6 g	0.6 g	0.6 g	0.6 g
pH	5.8	5.8	5.8	5.8	5.8
Acetosyringone stock solution (20 mg/ml)	1 ml	1 ml	0	0	0
Plant Agar	0	7 g	7 g	7 g	7 g
Cefotaxime stock solution (300 mg/ml)	0	0	1 ml	1 ml	1 ml
Blp stock solution (25 mg/ml)	0	0	1 ml	1 ml	1 ml
2,4-D stock solution (2 mg/ml)	1 ml	1 ml	1 ml	0	0
6-BA stock solution (0.5 mg/ml)	1 ml	1 ml	1 ml	2 ml	0
NAA stock solution (0.1 mg/ml)	0	0	0	5 ml	1 ml
KT stock solution (1 mg/ml)	0	0	0	1 ml	0
